# Discrimination of Non-Local Correlations

**DOI:** 10.3390/e21020104

**Published:** 2019-01-23

**Authors:** Alberto Montina, Stefan Wolf

**Affiliations:** Facoltà di Informatica, Università della Svizzera italiana, 6900 Lugano, Switzerland

**Keywords:** local polytope, quantum nonlocality, communication complexity, optimization

## Abstract

In view of the importance of quantum non-locality in cryptography, quantum computation, and communication complexity, it is crucial to decide whether a given correlation exhibits non-locality or not. As proved by Pitowski, this problem is NP-complete, and is thus computationally intractable unless NP is equal to P. In this paper, we first prove that the Euclidean distance of given correlations from the local polytope can be computed in polynomial time with arbitrary fixed error, granted the access to a certain oracle; namely, given a fixed error, we derive two upper bounds on the running time. The first bound is linear in the number of measurements. The second bound scales with the number of measurements to the sixth power. The former holds only for a very high number of measurements, and is never observed in the performed numerical tests. We, then, introduce a simple algorithm for simulating the oracle. In all of the considered numerical tests, the simulation of the oracle contributes with a multiplicative factor to the overall running time and, thus, does not affect the sixth-power law of the oracle-assisted algorithm.

## 1. Introduction

Non-local correlations, displayed by certain entangled quantum systems, mark a clear departure from the classical framework made up of well-defined, locally interacting quantities [1]. Besides their importance in foundation of quantum theory, non-local correlations have gained interest as information-processing resources in cryptography [2,3,4,5,6,7,8], randomness amplification [9,10], quantum computation, and communication complexity [11]. In view of their importance, a relevant problem— hereafter called the *non-locality problem*—is to find a criterion for deciding if observed correlations are actually non-local. Such a criterion is, for example, provided by the *Bell inequalities* [12]. However, a result by Pitowski [12] suggests that the problem of discriminating between local and non-local correlations is generally intractable. Pitowski proved that deciding membership to the correlation polytope is NP-complete, and is therefore intractable unless NP is equal to P. This result also implies that the opposite problem, deciding whether given correlations are outside the polytope, is not even in NP, unless NP=co-NP—which is believed to be false.

In this paper, we present an algorithm whose numerical tests suggest a polynomial running time for all the considered quantum-correlation problems. More precisely, the algorithm computes the distance from the local polytope. First, we prove that the time cost of computing the distance with an arbitrary fixed error grows polynomially in the size of the problem input (number of measurements and outcomes), granted the access to a certain oracle. Namely, given a fixed error, we derive two upper bounds on the running time. The first bound is linear in the number of measurements. The second bound scales with the number of measurements to the sixth power. The former holds only for a very high number of measurements, and is never observed in the performed numerical tests. Thus, the problem of computing the distance is reduced to determining an efficient simulation of the oracle. Then, we introduce a simple algorithm that simulates the oracle. The algorithm is probabilistic and provides the right answer in a subset of randomized inputs. Thus, to have a correct answer with sufficiently high probability, the simulation of the oracle has to be performed with a suitably high number of initial random inputs. In the numerical tests, the number of random initial trials has pragmatically been chosen such that the simulation of the oracle contributes to the overall running time with a multiplicative factor and, thus, does not affect the sixth-power law of the oracle-assisted algorithm. In all of the performed numerical tests, the overall algorithm always computes the distance within the desired accuracy. The scaling of the running time observed in the tests is compatible with the sixth-power law, derived theoretically.

Similar results have independently been published in [13], almost simultaneously to a first version of this paper [14]. The algorithm in [13] is a modification of Gilbert’s algorithm for minimizing quadratic forms in a convex set. In its original form, the algorithm uses the following strategy for generating a sequence of points, which converge to the minimizer: Given a point $P_{n}$ of the sequence, a procedure of linear optimization generates another point $Q_{n}$, such that the next point $P_{n+1}$ of the sequence is computed as a convex combination of $P_{n}$ and $Q_{n}$. If the convex set is a polytope, the points $Q_{1},\cdots$ turn out to be vertices of the polytope. The modified algorithm, introduced in [13], keeps track of the previous vertices $Q_{n-m},Q_{n-m+1},\cdots ,Q_{n}$, *m* being some fixed parameter, and computes the next point $P_{n+1}$ as convex combination of these points and $P_{n}$. In our algorithm, we compute $P_{n+1}$ as a convex combination of a suitable set of previously computed vertices, without using the point $P_{n}$ (Section 5). This difference does not result in substantial computational differences. However, our approach has the advantage of keeping track of the minimal number of vertices required for a convex representation of the optimizer. In particular, in the case of local correlations, the algorithm immediately gives a minimal convex representation of them. This representation provides a certificate, which another party can use for directly proving locality. As another minor difference, our algorithm actually computes the distance from what we will call the *local cone*. This allows us to eliminate a normalization constraint from the optimization problem.

The paper is organized as follows. In Section 2, we introduce our general scenario. For the sake of simplicity, we will discuss only the two-party case, but the results can be extended to the general case of many parties. After introducing the local polytope in Section 3, we formulate the non-locality problem as a minimization problem; namely, the problem of computing the distance from the local polytope (Section 4). In Section 5, the algorithm is introduced. The convergence and the computational cost are then discussed in Section 6. After introducing the algorithm for solving the oracle, we finally discuss the numerical results in Section 7.

## 2. Nonsignaling Box

In a Bell scenario, two quantum systems are prepared in an entangled state and delivered to two spatially separate parties; say, Alice and Bob. These parties each perform a measurement on their system and get an outcome. In general, Alice and Bob are allowed to choose among their respective sets of possible measurements. We assume that the sets are finite, but arbitrarily large. Let us denote the measurements performed by Alice and Bob by the indices a∈{1,⋯,A} and b∈{1,⋯,B}, respectively. After the measurements, Alice gets an outcome r∈R and Bob an outcome s∈S, where R and S are two sets with cardinality *R* and *S*, respectively. The overall scenario is described by the joint conditional probability P(r,s|a,b) of getting (r,s), given (a,b). Since the parties are spatially separate, causality and relativity imply that this distribution satisfies the nonsignaling conditions

(1) $$\begin{array}{c} P\left(r|a,b\right)=P\left(r|a,\bar{b}\right)\;\;\forall r,a,b,\bar{b},\;\mathrm{and}\;\\ P\left(s|a,b\right)=P\left(s|\bar{a},b\right)\;\;\forall s,b,a,\bar{a}, \end{array}$$

where $P\left(r|a,b\right)\equiv \sum_{s}P\left(r,s|a,b\right)$ and $P\left(s|a,b\right)\equiv \sum_{r}P\left(r,s|a,b\right)$ are the marginal conditional probabilities of *r* and *s*, respectively. In the following discussion, we consider a more general scenario than quantum correlations, and we just assume that P(r,s|a,b) satisfies the nonsignaling conditions. The abstract machine producing the correlated variables *r* and *s* from the inputs *a* and *b* will be called the *nonsignaling box* (briefly, NS-box).

## 3. Local Polytope

The correlations between the outcomes *r* and *s*, associated with the measurements *a* and *b*, are *local* if and only if the conditional probability P(r,s|a,b) can be written in the form

(2) $$P\left(r,s|a,b\right)=\sum_{x}P^{A}\left(r|a,x\right)P^{B}\left(s|b,x\right)P^{S}\left(x\right),$$

where $P^{A}$, $P^{B}$, and $P^{S}$ are suitable probability distributions. It is always possible to write the conditional probabilities $P^{A}$ and $P^{B}$ as convex combination of local deterministic processes, that is,

(3) $$\begin{array}{c} P^{A}\left(r|a,x\right)=\sum_{r}P_{\det}^{A}\left(r|r,a\right)\rho^{A}\left(r|x\right),\;\mathrm{and}\;\\ P^{B}\left(s|b,x\right)=\sum_{s}P_{\det}^{B}\left(s|s,b\right)\rho^{B}\left(s|x\right), \end{array}$$

where $r\equiv (r_{1},\cdots ,r_{A})$, $s\equiv (s_{1},\cdots ,s_{B})$, $P_{\det}^{A}\left(r|r,a\right)=\delta_{r_{a},r}$, and $P_{\det}^{B}\left(s|s,b\right)=\delta_{s_{b},s}$. Using this decomposition, Equation (2) takes the form of a convex combination of local deterministic distributions. That is,

(4) $$\begin{array}{ccc} P(r,s|a,b) & = & \sum_{r,s}P_{\det}^{A}\left(r|r,a\right)P_{\det}^{B}\left(s|s,b\right)P^{AB}\left(r,s\right)\\ & = & \sum_{r,s}\delta_{r,r_{a}}\delta_{s,s_{b}}P^{AB}\left(r,s\right)\\ & = & \sum_{r,r_{a}=r}\sum_{s,s_{b}=s}P^{AB}\left(r,s\right), \end{array}$$

where $P^{AB}\left(r,s\right)\equiv \sum_{x}\rho^{A}\left(r|x\right)\rho^{B}\left(s|x\right)P^{S}\left(x\right)$ and $\delta_{i,j}$ is the Kronecker delta. Equation (5) is known as Fine’s theorem [15]. Thus, a local distribution can always be written as convex combination of local deterministic distributions. Clearly, the converse is also true and a convex combination of local deterministic distributions is local. Therefore, the set of local distributions is a polytope, called a *local polytope*. As the deterministic probability distributions $P_{\det}^{A}\left(r|r,a\right)P_{\det}^{B}\left(s|s,b\right)$ are not convex combinations of other distributions, they all define the vertices of the local polytope. Thus, there are $R^{A}S^{B}$ vertices, each one specified by the sequences r and s. Let us denote the map from (r,s) to the associated vertex by $\vec{V}$. That is, $\vec{V}$ maps the sequences to a deterministic local distribution,

(5) $$\vec{V}\left(r,s\right)\equiv P_{det}:\left(r,s,a,b\right)\mapsto \delta_{r,r_{a}}\delta_{s,s_{b}}.$$

Since the elements of the local polytope are normalized distributions and satisfy the nonsignaling conditions (1), the RSAB parameters defining P(r,s|a,b) are not independent and the polytope lives in a lower-dimensional subspace. The dimension of this subspace and, more generally, of the subspace of NS-boxes, is equal to [16]

(6) $$d_{NS}\equiv AB\left(R-1\right)\left(S-1\right)+A\left(R-1\right)+B\left(S-1\right).$$

By the Minkowski–Weyl theorem, the local polytope can be represented as the intersection of finitely many half-spaces. A half-space is defined by an inequality

(7) $$\sum_{r,s,a,b}P\left(r,s|a,b\right)B\left(r,s;a,b\right)\leq L.$$

In the case of the local polytope, these inequalities are called *Bell inequalities*. Given the coefficients B(r,s;a,b), we can choose *L* such that the inequality is as restrictive as possible. This is attained by imposing that at least one vertex of the local polytope is at the boundary of the half-space; that is, by taking

(8) $$L=\max_{r,s}\sum_{a,b}B\left(r_{a},s_{b};a,b\right).$$

The oracle, which is central in this work, and introduced later in Section 4, returns the value *L* from the coefficients B(r,s;a,b).

A minimal representation of a polytope is given by the set of facets of the polytope. A half-space $\sum_{r,s,a,b}P\left(r,s|a,b\right)B\left(r,s;a,b\right)\leq L$ specifies a facet if the associated hyperplane $\sum_{r,s,a,b}P\left(r,s|a,b\right)B\left(r,s;a,b\right)=L$ intersects the boundary of the polytope in a set with dimension equal to the dimension of the polytope minus one. A distribution P(r,s|a,b) is local if and only if every facet inequality is not violated. Deciding whether some inequality is violated is generally believed to be intractable, due to a result by Pitowski [12], but to test the membership of a distribution to the local polytope can be done in polynomial time, once the vertices—of which the distribution is a convex combination—are known. Thus, deciding membership to the local polytope is an NP problem. Furthermore, the problem is NP-complete [12].

## 4. Distance from the Local Polytope

The non-locality problem can be reduced to a convex optimization problem, such as the computation of the nonlocal capacity, introduced in [17], and the distance from the local polytope, which can be reduced to a linear program if the $L^{1}$ norm is employed [18]. Here, we define the distance of a distribution P(r,s|a,b) from the local polytope as the Euclidean distance between P(r,s|a,b) and the closest local distribution. As mentioned in Section 3 (see Equation (5)), and stated by Fine’s theorem [15], a conditional distribution ρ(r,s|a,b) is local if and only if there is a non-negative function χ(r,s) such that

(9) $$\rho \left(r,s|a,b\right)=\sum_{r,r_{a}=r}\sum_{s,s_{b}=s}\chi \left(r,s\right).$$

That is, a conditional distribution ρ(r,s|a,b) is local if it is the marginal of a multivariate probability distribution χ of the outcomes of all the possible measurements, provided that χ does not depend on the measurements *a* and *b*.

The distributions P(r,s|a,b) and ρ(r,s|a,b) can be represented as vectors in a RSAB-dimensional space. Let us denote them by $\vec{P}$ and $\vec{\rho }$, respectively. Given a positive-definite matrix $\hat{M}$ defining the metrics in the vector space, the computation of the distance from the local polytope is equivalent to the minimization of a functional of the form

(10) $$F\left[\chi \right]=\frac{1}{2}{\left(\vec{P}-\vec{\rho }\right)}^{T}\hat{M}\left(\vec{P}-\vec{\rho }\right)$$

with respect to χ, under the constraints that χ is non-negative and normalized. Namely, the distance is the square root of the minimum of 2F. Hereafter, we choose the metrics so that the functional takes the form

(11) $$F\left[\chi \right]\equiv \frac{1}{2}\sum_{r,s,a,b}{\left[P(r,s|a,b)-\rho (r,s|a,b)\right]}^{2}W\left(a,b\right),$$

where W(a,b) is some probability distribution. The normalization $\sum_{a,b}W\left(a,b\right)=1$ guarantees that the distance does not diverge in the limit of infinite measurements performed on a given entangled state. In particular, we will consider the case with

(12) $$W\left(a,b\right)\equiv \frac{1}{AB}.$$

Another choice would be to take the distribution W(a,b) maximizing the functional, so that the computation of the distance would be a minimax problem. This case has some interesting advantages, but is more sophisticated and will not be considered here. Since we are interested in a quantity that is equal to zero if and only if P(r,s|a,b) is local, we can simplify the problem of computing the distance by dropping the normalization constraint on χ. Indeed, if the distance is equal to zero, ρ, and thus χ, are necessarily normalized. Conversely, if the distance is different from zero for every normalized local distribution, it is so also for every unnormalized local distribution. Thus, the discrimination between local and non-local correlation is equivalent to the following minimization problem.

**Problem** **1.** $$\begin{array}{c} \min_{\chi }F\left[\chi \right]\\ \mathrm{subject}\;\mathrm{to}\;\mathrm{the}\;\mathrm{constraints}\\ \chi (r,s)\geq 0. \end{array}$$

Let us denote the solution of this problem and the corresponding optimal value by $\chi^{min}$ and $F^{min}$, respectively. The associated (unnormalized) local distribution is denoted by $\rho^{min}\left(r,s|a,b\right)$. The square root of $2F^{min}$ is the minimal distance of P(r,s|a,b) from the cone defined as the union of all the lines connecting the zero distribution ρ(r,s|a,b)=0 and an arbitrary point of the local polytope. Let us call this set the *local cone*. Hereafter, we will consider the problem of computing the distance from the local cone, but the results can be easily extended to the case of the local polytope, so that we will use “local cone” and “local polytope” as synonyms in the following discussion. Note that there are generally infinite minimizers $\chi^{min}$, since χ lives in a $R^{A}S^{B}$-dimensional space, whereas the functional *F* depends on χ through ρ(r,s|a,b), which lives in a $\left(d_{NS}+1\right)$-dimensional space. In other words, since the local polytope has $R^{A}S^{B}$ vertices, but the dimension of the polytope is $d_{NS}$, a (normalized) distribution ρ has generally infinite representations as convex combination of the vertices, unless ρ is on a face whose dimension plus 1 is equal to the number of vertices defining the face.

At first glance, the computational complexity of this problem seems intrinsically exponential, as the number of real variables defining χ is equal to $R^{A}S^{B}$. However, the dimension of the local polytope is $d_{NS}$ and grows polynomially in the number of measurements and outcomes. Thus, by Carathéodory’s theorem, a (normalized) local distribution can always be represented as the convex combination of a number of vertices smaller than $d_{NS}+2$. This implies that there is a minimizer $\chi^{min}$ of *F* whose support contains a number of elements not greater than $d_{NS}+1$. Therefore, the minimizer can be represented by a number of variables growing polynomially in the input size. The main problem is to find a small set of vertices that are suitable for representing the closest local distribution $\rho^{min}\left(r,s|a,b\right)$. In the following, we will show that the computation of the distance from the local cone with an arbitrary fixed accuracy has polynomial complexity, granted the access to the following oracle.

*Oracle Max*: Given a function g(r,s;a,b), the oracle returns the sequences r and s maximizing the function

(13) $$G\left(r,s\right)\equiv \sum_{a,b}g\left(r_{a},s_{b};a,b\right)W\left(a,b\right)$$

and the corresponding maximal value.

Thus, Problem 1 is reduced to determining an efficient simulation of the oracle. Let us consider the case of binary outcomes, with *r* and *s* taking values ±1 (R=S=2). The function G(r,s) takes the form

(14) $$G\left(r,s\right)=\sum_{a,b}J_{ab}r_{a}s_{b}+\sum_{a}A_{a}r_{a}+\sum_{b}B_{b}s_{b}+G_{0},$$

whose minimization falls into the class of spin-glass problems, which are notoriously computationally hard to handle. This suggests that the oracle is generally an intractable problem. Nonetheless, the oracle has a particular structure that can make the problem easier to be solved, in some instances. This will be discussed later, in Section 6.3 and Section 7. There, we will show that the oracle can be simulated efficiently in many relevant cases, by using a simple block-maximization strategy. Assuming for the moment that we have access to the oracle, let us introduce the algorithm solving Problem 1.

## 5. Computing the Distance

The distance from the local polytope can be computed efficiently, once we have a set Ω of vertices that is small enough and suitable for representing the closest distribution $\rho^{min}\left(r,s|a,b\right)$. The algorithm introduced in this paper solves Problem 1 by iteratively generating a sequence of sets Ω. At each step, the minimal distance is first computed over the convex hull of the given vertices. Then, the oracle is consulted. If the set does not contain the right vertices, the oracle returns a strictly positive maximal value and a vertex, which is added to the set Ω (after possibly removing vertices with zero weight). The optimization Problem 1 is solved once the oracle returns zero, which guarantees that all the optimality conditions of the problem are satisfied. Before discussing the algorithm, let us derive these conditions.

### 5.1. Necessary and Sufficient Conditions for Optimality

Problem 1 is a convex optimization problem whose constraints satisfy Slater’s condition, requiring the existence of an interior point of the feasible region. This is the case, as a positive χ strictly satisfies all the inequality constraints. Thus, the four Karush–Kuhn–Tucker (KKT) conditions are necessary and sufficient conditions for optimality. Let us briefly summarize these conditions. Given an objective function $F(\vec{x})$ of the variables $\vec{x}$ and equality constraints $G_{k=1,\cdots ,n_{c}}\left(\vec{x}\right)=0$, it is well known that the function *F* is stationary at $\vec{x}$ if the gradient of the Lagrangian $L\left(\vec{x}\right)\equiv F\left(\vec{x}\right)-\sum_{k=1}^{n_{c}}\eta_{k}G_{k}\left(\vec{x}\right)$ is equal to zero, for some value of the Lagrange multipliers $\eta_{k}$. This is the first KKT condition. The second condition is the feasibility of the constraints; that is, the stationary point $\vec{x}$ must satisfy the constraints $G_{k}\left(\vec{x}\right)=0$. These two conditions are necessary and sufficient, as there are only equality constraints. If there are also inequalities, two additional conditions on the associated Lagrange multipliers are required. Given inequality constraints $H_{k}\left(\vec{x}\right)\geq 0$, with associated Lagrange multipliers $\lambda_{k}$, the third condition is the non-negativity of the multipliers; that is, $\lambda_{k}\geq 0$. This condition says that the constraint acts only in one direction, like a floor acts on objects through an upward force, but not with a downward force. The last condition states that the Lagrange multiplier $\lambda_{k}$ can differ from zero only if the constraint is active; that is, if $H_{k}\left(\vec{x}\right)=0$. This is like stating that a floor acts on a body only if they are touching (contact force). This condition can concisely be written as $\lambda_{k}H_{k}\left(\vec{x}\right)=0$.

Let us characterize the optimal solution of Problem 1 through the four KKT conditions.

- First KKT condition (*stationarity condition*): The gradient of the Lagrangian is equal to zero. The Lagrangian of Problem 1 is
  (15) $$L=F\left[\chi \right]-\sum_{r,s}\lambda \left(r,s\right)\chi \left(r,s\right),$$
  where λ(r,s) are the Lagrange multipliers associated with the inequality constraints.
- Second KKT condition (*feasibility of the constraints*): The function χ is non-negative, χ(r,s)≥0.
- Third condition (*dual feasibility*): The Lagrange multipliers λ are non-negative; that is,
  (16) λ(r,s)≥0.
- Fourth condition (*complementary slackness*): If χ(r,s)≠0, then the multiplier λ(r,s) is equal to zero; that is,
  (17) λ(r,s)χ(r,s)=0.

The stationarity condition on the gradient of the Lagrangian gives the equality

(18) $$\sum_{a,b}W\left(a,b\right)\left[P\left(r_{a},s_{b}|a,b\right)-\rho \left(r_{a},s_{b}|a,b\right)\right]+\lambda \left(r,s\right)=0.$$

Eliminating λ, this equality and the dual feasibility yield the inequality

(19) $$\sum_{a,b}W\left(a,b\right)\left[P\left(r_{a},s_{b}|a,b\right)-\rho \left(r_{a},s_{b}|a,b\right)\right]\leq 0.$$

From Equation (18), we have that the complementary slackness is equivalent to the following condition,

(20) $$\begin{array}{c} \chi (r,s)\neq 0\Rightarrow \;\\ \sum_{a,b}W\left(a,b\right)\left[P\left(r_{a},s_{b}|a,b\right)-\rho \left(r_{a},s_{b}|a,b\right)\right]=0; \end{array}$$

that is, the left-hand side of the last inequality is equal to zero if (r,s) is in the support of χ. The slackness condition (20), the primal constraint and Equation (19) provide necessary and sufficient conditions for optimality. Let us introduce the function

(21) g(r,s;a,b)≡P(r,s|a,b)−ρ(r,s|a,b),

which is the opposite of the gradient of *F* with respect to ρ, up to the factor W(a,b). Summarizing, the conditions are

(22) $$\begin{array}{cc} & \sum_{a,b}W\left(a,b\right)g\left(r_{a},s_{b};a,b\right)\leq 0, \end{array}$$

(23) $$\begin{array}{cc} & \chi \left(r,s\right)\neq 0\Rightarrow \sum_{a,b}W\left(a,b\right)g\left(r_{a},s_{b};a,b\right)=0, \end{array}$$

(24) $$\begin{array}{cc} & \chi (r,s)\geq 0. \end{array}$$

The second condition can be rewritten in the more concise form

(25) $$\sum_{r,s,a,b}\rho \left(r,s|a,b\right)g\left(r,s|a,b\right)W\left(a,b\right)=0.$$

Indeed, using Equations (22) and (24), it is easy to show that condition (23) is satisfied if and only if

$$\sum_{r,s}\chi \left(r,s\right)\sum_{a,b}W\left(a,b\right)g\left(r_{a},s_{b};a,b\right)=0,$$

which gives equality (25), by definition of ρ (Equation (9)).

Condition (22) can be checked, by consulting the oracle with g(r,s;a,b) as the query. If the oracle returns a non-positive maximal value, then the condition is satisfied. Actually, at the optimal point, the returned value turns out to be equal to zero, as implied by the other optimality conditions.

Similar optimality conditions hold if we force χ to be equal to zero outside some set Ω. Let us introduce the following minimization problem.

**Problem** **2.** $$\begin{array}{c} \min_{\chi }F\left[\chi \right]\\ \mathrm{subject}\;\mathrm{to}\;\mathrm{the}\;\mathrm{constraints}\\ \chi (r,s)\geq 0,\\ \chi (r,s)=0\;\;\;\forall (r,s)\notin \Omega . \end{array}$$

The optimal value of this problem gives an upper bound on the optimal value of Problem 1. The two problems are equivalent if the support of a minimizer $\chi^{min}$ of Problem 1 is in Ω. The necessary and sufficient conditions for optimality of Problem 2 are the same as of Problem 1, with the only difference that condition (22) has to hold only in the set Ω. That is, the condition is replaced by the weaker condition

(26) $$\left(r,s\right)\in \Omega \Rightarrow \sum_{a,b}W\left(a,b\right)g\left(r_{a},s_{b};a,b\right)\leq 0.$$

Thus, an optimizer of Problem 2 is solution of Problem 1 if the value returned by the oracle with query g=P−ρ is equal to zero.

Hereafter, the minimizer and the minimal value of Problem 2 will be denoted by $\chi_{\Omega }^{min}$ and $F_{\Omega }^{min}$, respectively. The associated optimal local distribution ρ(r,s|a,b), defined by Equation (9), will be denoted by $\rho_{\Omega }^{min}\left(r,s|a,b\right)$.

### 5.2. Overview of the Algorithm

Problem 1 can be solved iteratively by finding the solution of Problem 2 over a sequence of sets Ω. The sets are built according to the answer of the oracle, which is consulted at each step of the iteration. The procedure stops when a desired accuracy is reached or Ω contains the support of a minimizer $\chi^{min}$, and the solution of Problem 2 is also the solution of Problem 1. Let us outline the algorithm. Suppose that we choose the initial Ω as a set of sequences (r,s) associated to $n_{0}$ linearly independent vertices ($n_{0}$ being possibly equal to 1). Let us denote this set by $\Omega_{0}$. We solve Problem 2 with $\Omega =\Omega_{0}$ and get the optimal value $F_{0}^{min}\equiv F_{\Omega_{0}}^{min}$ with minimizer $\chi_{0}^{min}\equiv \chi_{\Omega_{0}}^{min}$. Let us denote the corresponding (unnormalized) local distribution by $\rho_{0}^{min}\equiv \rho_{\Omega_{0}}^{min}$. That is,

(27) $$\rho_{0}^{min}\left(r,s|a,b\right)\equiv \sum_{r,r_{a}=r}\sum_{s,s_{b}=s}\chi_{0}^{min}\left(r,s\right).$$

Since the cardinality of $\Omega_{0}$ is not greater than $d_{NS}+1$ and the problem is a convex quadratic optimization problem, the corresponding computational complexity is polynomial. Generally, a numerical algorithm provides an optimizer, up to some arbitrarily small but finite error. In Section 5.5, we will provide a bound on the accuracy required for the solution of Problem 2. For now, let us assume that Problem 2 is solved exactly. If the support of $\chi^{min}$ is in $\Omega_{0}$, $F_{0}^{min}$ is equal to the optimal value of Problem 1, and we have computed the distance from the local polytope. We can verify if this is the case by checking the first optimality condition (22), as the conditions (23) and (24) are trivially satisfied by the optimizer of Problem 2 for every (r,s). The check is made by consulting the oracle with the function $P\left(r,s|a,b\right)-\rho_{0}^{min}\left(r,s|a,b\right)$ as the query. If the oracle returns a maximal value equal to zero, then we have the solution of Problem 1. Note that if the optimal value of Problem 2 is equal to zero, then also the optimal value of the main problem is equal to zero and the conditional distribution P(r,s|a,b) is local. In this case, we have no need of consulting the oracle.

If the optimal value of Problem 2 is different from zero and the oracle returns a maximal value strictly positive, then the minimizer of Problem 2 satisfies all the optimality conditions of Problem 1, except Equation (22) for some (r,s)∉Ω. The next step is to add the pair of sequences (r,s) returned by the oracle to the set Ω and solve Problem 2 with the new set. Let us denote the new set and the corresponding optimal value by $\Omega_{1}$ and $F_{1}^{min}\equiv F_{\Omega_{1}}^{min}$, respectively. Once we have solved Problem 2 with $\Omega =\Omega_{1}$, we consult again the oracle to check if we have obtained the solution of Problem 1. If we have not, we add the pair of sequences (r,s) given by the oracle to the set Ω and we solve Problem 2 with the new set, say $\Omega_{2}$. We continue until we get the solution of Problem 1 or its optimal value up to some desired accuracy. This procedure generates a sequence of sets $\Omega_{n=1,2,\cdots }$ and values $F_{n=1,2,\cdots }^{min}$. The latter sequence is strictly decreasing, that is, $F_{n+1}^{min}<F_{n}^{min}$ until $\Omega_{n}$ contains the support of $\chi^{min}$ and the oracle returns zero as maximal value. Let us show that. Suppose that $\chi_{n}^{min}$ is the optimizer of Problem 2 with $\Omega =\Omega_{n}$ and $\left(r^{\prime },s^{\prime }\right)$ is the new element in the set $\Omega_{n+1}$. Let us denote by $\rho_{n}^{min}\left(r,s|a,b\right)$ the local distribution associated with $\chi_{n}^{min}$, that is,

(28) $$\rho_{n}^{min}\left(r,s|a,b\right)\equiv \sum_{r,r_{a}=r}\sum_{s,s_{b}=s}\chi_{n}^{min}\left(r,s\right).$$

The optimal value $F_{n+1}^{min}$ of Problem 2 is bounded from above by the value taken by the function F[χ] for every feasible χ, in particular, for

(29) $$\chi \left(r,s;\alpha \right)=\chi_{n}^{min}\left(r,s\right)+\alpha \delta_{r,r^{\prime }}\delta_{s,s^{\prime }},$$

with α positive. Let us set α equal to the value minimizing *F*; that is,

(30) $$\alpha \equiv \alpha_{n}=\sum_{ab}W\left(a,b\right)\left[P\left(r_{a}^{\prime },s_{b}^{\prime }|a,b\right)-\rho_{n}^{min}\left(r_{a}^{\prime },s_{b}^{\prime }|a,b\right)\right],$$

which is equal to the value returned by the oracle. It is strictly positive, as the oracle returned a positive value—provided that $\Omega_{n}$ does not contain the support of $\chi^{min}$. Hence, $\chi (r,s;\alpha_{n})$ is a feasible point and, thus, the corresponding value taken by *F*,

(31) $${\left.F\right|}_{\alpha =\alpha_{n}}=F_{n}^{min}-\frac{1}{2}\alpha_{n}^{2},$$

is an upper bound on $F_{n+1}^{min}$. Hence,

(32) $$F_{n+1}^{min}\leq F_{n}^{min}-\frac{1}{2}\alpha_{n}^{2},$$

that is, $F_{n+1}^{min}$ is strictly smaller than $F_{n}^{min}$.

This procedure generates a sequence $F_{n}^{min}$ that converges to the optimal value of Problem 1, as shown in Section 6. For any given accuracy, the computational cost of the procedure is polynomial, provided that we have access to the oracle.

To avoid growth of the cardinality of Ω beyond $d_{NS}+1$ during the iteration and, thus, the introduction of redundant vertices, we have to be sure that the sets $\Omega_{0},\Omega_{1},\cdots$ contain points (r,s) associated to linearly independent vertices $\vec{V}\left(r,s\right)$ of the local polytope. This is guaranteed by the following procedure of cleaning up. First, after the computation of $\chi_{n}^{min}$ at step *n*, we remove the elements in $\Omega_{n}$ where $\chi_{n}^{min}\left(r,s\right)$ is equal to zero (this can be checked even if the exact $\chi_{n}^{min}$ is not known, as discussed later in Section 5.6). Let us denote the resulting set by $\Omega_{n}^{clean}$. Then, the set $\Omega_{n+1}$ is built by adding the point given by the oracle to the set $\Omega_{n}^{clean}$. Let us denote by V the set of vertices associated to the elements in the support of $\chi_{n}^{min}$. The cleaning up ensures that the optimizer $\rho_{n}^{min}$ is in the interior of the convex hull of V, up to a normalization constant, and the new vertex returned by the oracle is linearly independent of the ones in V. Indeed, we have seen that the introduction of such a vertex allows us to lower the optimal value of Problem 2. This would not be possible if the added vertex was linearly dependent on the vertices in V, as the (normalized) optimizer $\rho_{n}^{min}$ of Problem 2 is in the interior of the convex hull of V.

This is formalized in Lemma 1.

**Lemma** **1.** *Let $\left(r^{\prime },s^{\prime }\right)$ be a sequence such that*

(33) $$\sum_{a,b}g\left(r_{a}^{\prime },s_{b}^{\prime };a,b\right)W\left(a,b\right)\neq 0.$$

*If *Ω* is a set such that*

(34) $$\left(r,s\right)=\Omega \Rightarrow \sum_{a,b}g\left(r_{a},s_{b};a,b\right)W\left(a,b\right)=0,$$

*then the vertex $\vec{V}\left(r^{\prime },s^{\prime }\right)$ is linearly independent of the vertices associated to the sequences in *Ω*.*

**Proof.**  The proof is by contradiction. Suppose that the vector $\vec{V}\left(r^{\prime },s^{\prime }\right)$ is linearly dependent with the vectors $\vec{V}\left(r,s\right)$ with (r,s)∈Ω, then there is a real function t(r,s) such that

(35) $$\vec{V}\left(r^{\prime },s^{\prime }\right)=\sum_{(r,s)\in \Omega }t\left(r,s\right)\vec{V}\left(r,s\right).$$

By definition of $\vec{V}$, this equation implies that $\sum_{r,s}t\left(r,s\right)\delta_{r,r_{a}}\delta_{s,s_{b}}=\delta_{r,r_{a}^{\prime }}\delta_{s,s_{b}^{\prime }}$. From this equation and Equation (34), we have

(36) $$\sum_{r,s}\delta_{r,r_{a}^{\prime }}\delta_{s,s_{b}^{\prime }}\sum_{a,b}g\left(r,s;a,b\right)W\left(a,b\right)=0.$$

Summing over *r* and *s*, we get a contradiction with Equation (33).  □

This lemma and the optimality conditions (22) and (23) imply that the sets $\Omega_{0},\Omega_{1},\cdots$, built through the previously discussed procedure of cleaning up, always contain points associated to independent vertices and, thus, never contain more than $d_{NS}+1$ elements. Indeed, the set $\Omega_{n}^{clean}$ contains points (r,s) where the minimizer $\chi_{n}^{min}$ is different from zero, for which

$$\sum_{a,b}\left[P\left(r_{a},s_{b}|a,b\right)-\rho_{n}^{min}\left(r_{a},s_{b}|a,b\right)\right]W\left(a,b\right)=0,$$

as implied by condition (23). Furthermore, given the sequence $\left(r^{\prime },s^{\prime }\right)$ returned by the oracle, condition (22) implies that

$$\sum_{a,b}\left[P\left(r_{a}^{\prime },s_{b}^{\prime }|a,b\right)-\rho_{n}^{min}\left(r_{a}^{\prime },s_{b}^{\prime }|a,b\right)\right]W\left(a,b\right)>0$$

until the set $\Omega_{n}$ contains the support of $\chi^{min}$ and the iteration generating the sequence of sets Ω is terminated.

The procedure of cleaning up is not strictly necessary for having a polynomial running time, but it can speed up the algorithm. Furthermore, the procedure guarantees that the distribution ρ(r,s|a,b) approaching the minimizer during the iterative computation is always represented as the convex combination of a minimal number of vertices. Thus, we have a minimal representation of the distribution at each stage of the iteration.

### 5.3. The Algorithm

In short, the algorithm for computing the distance from the local polytope with given accuracy is as follows.

**Algorithm** **1.** *Input: P(r,s|a,b)*

*1.*  *Set $\left(r^{\prime },s^{\prime }\right)$ equal to the sequences given by the oracle with P(r,s|a,b) as query.*
*2.*  *Set $\Omega =\{\left(r^{\prime },s^{\prime }\right)\}$.*
*3.*  *Compute the optimizers χ(r,s) and ρ(r,s|a,b) of Problem 2. The associated F provides an upper bound of the optimal value $F^{min}$.*
*4.*  *Consult the oracle with g(r,s;a,b)=P(r,s|a,b)−ρ(r,s|a,b) as query. Set $\left(r^{\prime },s^{\prime }\right)$ and α are equal to the sequences returned by the oracle and the associated maximal value, respectively. That is,*
  $$\left(r^{\prime },s^{\prime }\right)=\underset{\left(r,s\right)}{\mathrm{argmax}}\sum_{a,b}g\left(r_{a},s_{b};a,b\right)W\left(a,b\right),\;$$
  $$\alpha =\sum_{a,b}g\left(r_{a}^{\prime },s_{b}^{\prime }|a,b\right)W\left(a,b\right),$$
*5.*  *Compute a lower bound on the $F^{min}$ from ρ and α (see following discussion and Section 6.1). The difference between the upper and lower bounds provides an upper bound on the reached accuracy.*
*6.*  *If a given accuracy is reached, stop.*
*7.*  *Remove from *Ω* the points where χ is zero and add $\left(r^{\prime },s^{\prime }\right)$.*
*8.*  *Go back to Step 3.*

The algorithm stops at Step 6 when a desired accuracy is reached. To estimate the accuracy, we need to compute a lower bound on the optimal value $F^{min}$. To guarantee that the algorithm eventually stops, the lower bound has to converge to the optimal value as the algorithm approaches the solution of Problem 1. We also need a stopping criterion for the numerical routine solving the optimization problem in Step 3. Let us first discuss the stopping criterion for Algorithm 1.

### 5.4. Stopping Criterion for Algorithm 1

The lower bound on $F^{min}$, denoted by $F^{\left(-\right)}$, is computed by using the dual form of Problem 1. As shown in Section 6.1, any local distribution ρ induces the lower bound

(37) $$F^{\left(-\right)}=\frac{1}{2}\sum_{rsab}\{P^{2}\left(r,s|a,b\right)-[\rho \left(r,s|a,b\right)+\alpha ]{}^{2}\}W\left(a,b\right),$$

where α is the maximal value returned by the oracle with g(r,s;a,b)=P(r,s|a,b)−ρ(r,s|a,b) as query. An upper bound on $F^{min}$ is obviously

(38) $$F^{\left(+\right)}=F\left[\chi \right].$$

In the limit of ρ equal to the local distribution minimizing *F*, the lower bound is equal to the optimal value $F^{min}$. This can be shown by using the optimality conditions. Indeed, conditions (22) and (25) imply the limits

(39) $$\lim_{\chi \to \chi^{min}}\alpha =0,\;$$

(40) $$\lim_{\chi \to \chi^{min}}\sum_{r,s,a,b}\rho \left(r,s|a,b\right)g\left(r,s;a,b\right)W\left(a,b\right)=0,$$

which imply $F^{\left(-\right)}\to F^{min}$ as χ approaches the minimizer. This is made even more evident, by computing the difference between the upper bound and the lower bound. Indeed, given the local distribution ρ(r,s|a,b) computed at Step 3 and the corresponding α returned by the oracle at Step 4, the difference is

(41) $$\begin{array}{c} F^{\left(+\right)}-F^{\left(-\right)}\equiv \Delta F=\frac{RS}{2}\alpha^{2}+\;\\ \sum_{rsab}\rho \left(r,s|a,b\right)\left[\alpha -g(r,s;a,b)\right]W\left(a,b\right), \end{array}$$

which evidently goes to zero as χ goes to $\chi^{min}$. Thus, the upper bound ΔF on the accuracy computed in Step 5 goes to zero as ρ(r,s|a,b) approaches the solution. This guarantees that the algorithm stops sooner or later at Step 6, provided that χ converges to the solution. If Problem 2 is solved exactly at Step 3, then the distribution ρ(r,s|a,b) satisfies condition (25), and the upper bound on the reached accuracy takes the form

(42) $$F^{\left(+\right)}-F^{\left(-\right)}=\frac{RS}{2}\alpha^{2}+\alpha \sum_{rsab}\rho \left(r,s|a,b\right)W\left(a,b\right).$$

Even if Condition (25) is not satisfied, we can suitably normalize χ(r,s) so that the condition is satisfied.

In the following, we assume that this condition is satisfied.

### 5.5. Stopping Criterion for Problem 2 (Optimization at Step 3 of Algorithm 1)

In Algorithm 1, Step 3 is completed when the solution of Problem 2 with a given set Ω is found. Optimization algorithms iteratively find a solution $\rho_{\Omega }^{min}\left(r,s|a,b\right)$ up to some accuracy. We can stop when the error is of the order of the machine precision. Here, we will discuss a more effective stopping criterion. This criterion should preserve the two main features previously described:

- The sequence $F_{0}^{min},F_{1}^{min},\cdots$ of the exact optimal values of Problem 2, with $\Omega =\Omega_{0},\Omega_{1},\cdots$, is monotonically decreasing.
- The sets $\Omega_{0},\Omega_{1},\cdots$ contain points associated with linearly independent vertices of the local polytope, implying that the cardinality of $\Omega_{n}$ is never greater than $d_{NS}+1$.

To guarantee that the first feature is preserved, it is sufficient to compute a lower bound on $F_{\Omega }^{min}$ from a given χ so that the bound approaches $F_{\Omega }^{min}$ as χ approaches the optimizer $\chi_{\Omega }^{min}$. If the lower bound with the set $\Omega =\Omega_{n}$ is greater than the upper bound $F_{n}-\alpha_{n}^{2}/2$ on $F_{n+1}^{min}$ (see Equation (31)), then $F_{n+1}^{min}<F_{n}^{min}$. Denoting by $F_{\Omega }^{\left(-\right)}$ the lower bound on the optimal value $F_{\Omega }^{min}$, the monotonicity of the sequence $F_{0}^{min},F_{1}^{min},\cdots$ is implied by the inequality

(43) $$F_{n}-\frac{1}{2}\alpha_{n}^{2}\leq F_{\Omega_{n}}^{\left(-\right)}.$$

As shown later, by using dual theory, a lower bound on $F_{\Omega }^{min}$ is

(44) $$F_{\Omega }^{\left(-\right)}=\frac{1}{2}\sum_{rsab}\{P^{2}\left(r,s|a,b\right)-[\rho \left(r,s|a,b\right)+\beta ]{}^{2}\}W\left(a,b\right),$$

where

(45) $$\beta \equiv \max_{(r,s)\in \Omega }\sum_{ab}W\left(a,b\right)\left[P\left(r_{a},s_{b}|a,b\right)-\rho \left(r_{a},s_{b}|a,b\right)\right],$$

and ρ(r,s|a,b) is an unnormalized local distribution, associated to a function χ(r,s) with support in Ω. This bound becomes equal to $F_{\Omega }^{min}$ in the limit of ρ equal to the minimizer of Problem 2. Equation (43) gives the condition

(46) $$\begin{array}{c} \alpha^{2}>RS\beta^{2}+\;\\ 2\sum_{rsab}\left[\beta -g(r,s;a,b)\right]\rho \left(r,s|a,b\right)W\left(a,b\right), \end{array}$$

where g(r,s;a,b)=P(r,s|a,b)−ρ(r,s|a,b) and ρ(r,s|a,b) is the local distribution computed in Step 3. If this condition is satisfied by the numerical solution found in Step 3, then the series $F_{0}^{min},F_{1}^{min},\cdots$ is monotonically decreasing. As we will see, to prove that the series converges to the minimizer of Problem 1, we need the stronger condition

(47) $$\begin{array}{c} \gamma \alpha^{2}\geq RS\beta^{2}+\;\\ 2\sum_{rsab}\left[\beta -g(r,s;a,b)\right]\rho \left(r,s|a,b\right)W\left(a,b\right), \end{array}$$

where γ is any fixed real number in the interval (0,1). A possible choice is γ=1/2. If this inequality is satisfied in each iteration of Algorithm 1, the sequence $F_{0}^{min},F_{1}^{min},\cdots$ satisfies the inequality

(48) $$F_{n+1}^{min}\leq F_{n}^{min}-\frac{1-\gamma }{2}\alpha_{n}^{2},$$

which turns out to be equal to Equation (32) in the limit γ→0. The right-hand side of Equation (47) goes to zero as ρ approaches the optimizer, as implied by the optimality conditions of Problem 2. Thus, if the set Ω does not contain all the points where $\chi^{min}$ is different from zero, then the inequality is surely satisfied at some point of the iteration solving Problem 2, as α tends to a strictly positive number. When the inequality is satisfied, the minimization at Step 3 of Algorithm 1 is terminated. If Ω is the support of $\chi^{min}$, the inequality will never be satisfied and the minimization at Step 3 will terminate when the desired accuracy on $F^{min}$ is reached.

### 5.6. Cleaning Up (Step 7)

As previously said, we should also guarantee that the sets $\Omega_{n}$ contain only points associated with linearly independent vertices. This is granted if the procedure in Step 7 of Algorithm 1 successfully removes the points where the exact minimizer $\chi_{n}^{min}$ is equal to zero. How can we find the support of the minimizer from the approximate numerical solution computed in Step 3? Using dual theory, it is possible to prove the following.

**Theorem** **1.** *Let χ(r,s) be a non-negative function with support in *Ω* and ρ(r,s|a,b) be the associated unnormalized local distribution. Then, the inequality*

(49) $$\begin{array}{c} \sum_{a,b}\rho_{\Omega }^{min}\left(r_{a},s_{b}|a,b\right)W\left(a,b\right)\geq \sum_{a,b}\rho \left(r_{a},s_{b}|a,b\right)W\left(a,b\right)\\ -{\left[2\left(F^{\left(+\right)}-F_{\Omega }^{\left(-\right)}\right)\right]}^{1/2} \end{array}$$

*holds.*

A direct consequence of this theorem and the slackness condition (23) for optimality is the following.

**Corollary** **1.** *Let χ(r,s) be a non-negative function with support in *Ω* and ρ(r,s|a,b) the associated unnormalized local distribution. If the inequality*

(50) $$\begin{array}{c} \sum_{ab}g\left(r_{a},s_{b};a,b\right)\leq \left\{RS\beta^{2}+\right.\;\\ {\left.2\sum_{rsab}\left[\beta -g(r,s;a,b)\right]\rho \left(r,s|a,b\right)W\left(a,b\right)\right\}}^{1/2} \end{array}$$

*holds, with g(r,s|a,b)=P(r,s|a,b)−ρ(r,s|a,b), then $\chi_{\Omega }^{min}\left(r,s\right)$ is equal to zero.*

Condition (50) is sufficient for having $\chi_{\Omega }^{min}\left(r,s\right)$ equal to zero, but it is not necessary. A necessary condition can be derived by computing the lowest eigenvalue of the Hessian of the objective function F[χ]. Both the necessary and sufficient conditions allow us to determine the support of the minimizer $\chi_{\Omega }^{min}$ once the distribution χ is enough close to $\chi_{\Omega }^{min}$. Thus, the minimization in Step 3 should not stop until each sequence (r,s) satisfies the sufficient condition or does not satisfy the necessary condition, otherwise the cleaning up could miss some points where the minimizer is equal to zero. However, numerical experiments show that the use of these conditions is not necessary, and the number of elements in the sets $\Omega_{n}$ is generally bounded by $d_{NS}+1$, provided that Problem 2 is solved by the algorithm described in the following section.

### 5.7. Solving Problem 2

There are standard methods for solving Problem 2, and numerical libraries are available. The interior point method [19] provides a quadratic convergence to the solution, meaning that the number of digits of accuracy is almost doubled at each iteration step, once χ is sufficiently close to the minimizer. The algorithm uses the Newton method and needs to solve a set of linear equations. Since this can be computationally demanding in terms of memory, we have implemented the solver by using the conjugate gradient method, which does not use the Hessian. Furthermore, if the Hessian turns out to have a small condition number, the conjugate gradient method can be much more efficient than the Newton method, especially if we do not need to solve Problem 2 with high accuracy. This is the case in the initial stage of the computation, when the set Ω is growing and does not contain all the points of the support of $\chi^{min}$.

The conjugate gradient method iteratively performs a one-dimensional minimization, along directions that are conjugate with respect to the Hessian of the objective function [19]. The directions are computed iteratively, by setting the first direction equal to the gradient of the objective function. The conjugate gradient method is generally used with unconstrained problems, whereas Problem 2 has the inequality constraints χ(r,s)≥0. To adapt the method to our problem, we perform the one-dimensional minimization in the region where χ is non-negative. Whenever an inactive constraint becomes active, or vice versa, we set the search direction equal to the gradient and restart the generation of the directions from that point. Once the procedure terminates, the algorithm provides a list of active constraints with $\chi_{n}\left(r,s\right)=0$. Numerical simulations show that this list is generally complete, and corresponds to the points where the minimizer $\chi_{n}^{min}$ is equal to zero.

In general, the slackness condition (25) is not satisfied by the numerical solution. However, as previously pointed out, we can suitably normalize $\chi_{n}$ so that this condition is satisfied by $\rho_{n}\left(r,s|a,b\right)$. Thus, we will assume that the equality

(51) $$\sum_{r,s,a,b}\rho_{n}\left(r,s|a,b\right)g_{n}\left(r,s;a,b\right)W\left(a,b\right)=0$$

holds with $g_{n}=P-\rho_{n}$. This also implies that

(52) $$\begin{array}{c} \alpha_{n}={\left.\alpha \right|}_{\rho =\rho_{n}}\geq 0\;\mathrm{and}\\ \beta_{n}={\left.\beta \right|}_{\rho =\rho_{n}}\geq 0. \end{array}$$

## 6. Convergence Analysis and Computational Cost

Here, we provide a convergence analysis and show that the error on the distance from the local polytope is bounded above by a function decaying at least as fast as 1/n, where *n* is the number of iterations. The convergence of this function to zero is sublinear, but its derivation relies on a very rough estimate of a lower bound on the optimal value $\chi^{min}$. Actually, the iteration converges to the solution in a finite number of steps (up to the accuracy of the solver of Problem 2). Indeed, since the number of vertices is finite, also the number of their sets Ω is finite. Thus, the sequence $\Omega_{n}$ converges to the support of the optimizer $\chi^{min}$ in a finite number of steps, as the accuracy goes to zero.

We expect that this finite number of steps is of the order of the dimension $d_{NS}$ of the local polytope. Interestingly, the computed bound on the number of required iterations for given error does not depend on the number of measurements. Using this bound, we show that the computational cost for any given error on the distance grows polynomially with the size of the problem input; that is, with *A*, *B*, *R*, and *S*, provided that the oracle can be simulated in polynomial time.

To prove the convergence, we need to introduce the dual form of Problem 1 (see Ref. [19] for an introduction to dual theory). The dual form of a minimization problem (primal problem) is a maximization problem, whose maximum is always smaller than or equal to the primal minimum, the difference being called the *duality gap*. However, if the constraints of the primal problem satisfy some mild conditions, such as Slater’s conditions [19], then the duality gap is equal to zero. As previously said, this is the case of Problem 1.

The dual form is particularly useful for evaluating lower bounds on the optimal value of the primal problem. Indeed, the value taken by the dual objective function in a feasible point of the dual constraints provides such a bound. After introducing the dual form of Problem 1, we derive the lower bound $F^{\left(-\right)}$ on $F^{min}$, given by Equation (37). Then, we use this bound and Equation (48) to prove the convergence.

### 6.1. Dual Problem

The dual problem of Problem 1 is a maximization problem over the space of values taken by the Lagrange multipliers λ(r,s) subject to the dual constraints λ(r,s)≥0. The dual objective function is given by the minimum of the Lagrangian L, defined by Equation (15), with respect to χ. The dual constraint is the non-negativity of the Lagrange multipliers, that is,

(53) λ(r,s)≥0.

As this minimum cannot be derived analytically, a standard strategy for getting an explicit form of the dual objective function is to enlarge the space of primal variables and, correspondingly, to increase the number of primal constraints. The minimum is then evaluated over the enlarged space. In our case, it is convenient to introduce Equation (9) and ρ(r,s|a,b) as additional constraints and variables, respectively. Thus, *F* is made independent of χ and expressed as a function of ρ. The new optimization problem, which is equivalent to Problem 1, has Lagrangian

(54) $$\begin{array}{c} L=F\left[\rho \right]-\sum_{r,s}\lambda \left(r,s\right)\chi \left(r,s\right)+\sum_{rsab}W\left(a,b\right)\times \eta \left(r,s,a,b\right)\left[\rho \left(r,s|a,b\right)-\sum_{r,s}\delta_{r,r_{a}}\delta_{s,s_{b}}\chi \left(r,s\right)\right], \end{array}$$

where η(r,s,a,b) are the Lagrange multipliers associated with the added constraints. To find the minimum of the Lagrangian, we set its derivative, with respect to the primal variables χ and ρ, equal to zero. We get the equations

(55) $$\begin{array}{cc} & \sum_{a,b}W\left(a,b\right)\eta \left(r_{a},s_{b},a,b\right)=-\lambda \left(r,s\right) \end{array}$$

(56) $$\begin{array}{cc} \; & \rho (r,s|a,b)=P(r,s|a,b)-\eta (r,s,a,b). \end{array}$$

The first equation does not depend on the primal variables and sets a constraint on the dual variables. If this constraint is not satisfied, the dual objective function is equal to −∞. Thus, its maximum is in the region where Equation (55) is satisfied. Let us add it to the dual constraint (53). The second stationarity condition, Equation (56), gives the optimal ρ. By replacing it in the Lagrangian, we get the dual objective function

(57) $$F_{dual}=\sum_{r,s,a,b}W\left(a,b\right)\eta \left(r,s,a,b\right)\times \left[P\left(r,s|a,b\right)-\frac{\eta (r,s,a,b)}{2}\right].$$

Eliminating λ, which does not appear in the objective function, the dual constraints (53) and (55) give the inequality

(58) $$\sum_{a,b}W\left(a,b\right)\eta \left(r_{a},s_{b};a,b\right)\leq 0.$$

Thus, Problem 1 is equivalent to the following.

**Problem** **3** (dual problem of Problem 1). $$\begin{array}{c} \max_{\eta }F_{dual}\left[\eta \right]\\ \mathrm{subject}\;\mathrm{to}\;\mathrm{the}\;\mathrm{constraints}\\ \sum_{a,b}W\left(a,b\right)\eta \left(r_{a},s_{b};a,b\right)\leq 0. \end{array}$$

The value taken by $F_{dual}$ at a feasible point provides a lower bound on $F^{min}$. Given any function $\bar{\eta }\left(r,s;a,b\right)$, a feasible point is

(59) $$\eta_{f}\left(r,s;a,b\right)\equiv \bar{\eta }\left(r,s;a,b\right)-\max_{r,s}\sum_{\bar{a},\bar{b}}W\left(\bar{a},\bar{b}\right)\bar{\eta }\left(r_{\bar{a}},s_{\bar{b}};\bar{a},\bar{b}\right).$$

Indeed,

(60) $$\begin{array}{c} \sum_{a,b}\eta_{f}\left(r_{a},s_{b};a,b\right)W\left(a,b\right)=\sum_{a,b}\bar{\eta }\left(r_{a},s_{b};a,b\right)\;\\ -\max_{r^{\prime },s^{\prime }}\sum_{a,b}\bar{\eta }\left(r_{a}^{\prime },s_{b}^{\prime };a,b\right)W\left(a,b\right)\leq 0. \end{array}$$

The lower bound turns out to be the optimal value $F^{min}$, if the distribution ρ(r,s|a,b) given by Equation (56) in terms of $\eta =\eta_{f}$ is solution of the primal Problem 1. This suggests the transformation

(61) $$\bar{\eta }\left(r,s;a,b\right)=P\left(r,s|a,b\right)-\rho \left(r,s|a,b\right),$$

where ρ(r,s|a,b) is some local distribution up to a normalization constant (in fact, ρ can be any real function). Every local distribution induces a lower bound on the optimal value $F^{min}$. This lower bound turns out to be an accurate approximation of $F^{min}$ if ρ is close enough to the optimal local distribution. Using the last equation and Equation (59), we get the lower bound (37) from $F_{dual}$.

The dual problem of Problem 2 is similar to Problem 3, but the constraints have to hold for sequences (r,s) in Ω.

**Problem** **4** (dual problem of Problem 2). $$\begin{array}{c} \max_{\eta }F_{dual}\left[\eta \right]\\ \mathrm{subject}\;\mathrm{to}\;\mathrm{the}\;\mathrm{constraints}\\ \left(r,s\right)\in \Omega \Rightarrow \sum_{a,b}W\left(a,b\right)\eta \left(r_{a},s_{b};a,b\right)\leq 0. \end{array}$$

This dual problem induces the lower bound $F_{\Omega }^{min}$ on the optimal value of Problem 2 (Equation (44)).

### 6.2. Convergence and Polynomial Cost

Let $\rho_{n}\left(r,s|a,b\right)$ be the local distribution computed in Step 3 of Algorithm 1. From the lower bound (37), we have

(62) $$F^{min}\geq F_{n}-\frac{RS}{2}\alpha_{n}^{2}+\sum_{r,s,a,b}W\left(a,b\right)\rho_{n}\left(r,s|a,b\right)\left[g_{n}\left(r,s;a,b\right)-\alpha_{n}\right],$$

where $\alpha_{n}$ is given by Equation (30), and $g_{n}=P-\rho_{n}$. The part of the summation linear in $g_{n}$ is equal to zero, by Equation (51). The remaining part, linear in $\alpha_{n}$, is bounded from below by $-\alpha_{n}\left[1\;+\;{\left(RS\right)}^{1/2}\right]$ ($\alpha_{n}$ is positive). This can be shown by minimizing it under the constraint (51). Thus, we have that

(63) $$F^{min}\geq F_{n}-\frac{RS}{2}\alpha_{n}^{2}-\left[1+{\left(RS\right)}^{1/2}\right]\alpha_{n}.$$

As $\alpha_{n}$ is not greater than 1, the factor $\alpha_{n}^{2}$ in the right-hand side of the inequality can be replaced by $\alpha_{n}$, so that we have

(64) $$\alpha_{n}\geq 2\frac{F_{n}-F^{min}}{RS+2+2{\left(RS\right)}^{1/2}},$$

which gives, with Equation (48), the following

(65) $$F_{n}^{min}-F_{n+1}^{min}\geq 2\left(1-\gamma \right){\left(\frac{F_{n}-F^{min}}{RS+2+2{\left(RS\right)}^{1/2}}\right)}^{2}.$$

This inequality implies that

(66) $$F_{n}^{min}-F^{min}\leq \frac{{\left(RS+2+2{\left(RS\right)}^{1/2}\right)}^{2}}{2(1-\gamma )n}.$$

This can be proved by induction. It is easy to prove that inequality holds for $n=n_{0}>1$, if it holds for $n=n_{0}-1$. Let us prove that it holds for n=1. It is sufficient to prove that $F_{1}^{min}-F^{min}\leq 1/2$. Using the identity

(67) $$\sum_{r,s,a,b}W\left(a,b\right)\rho_{1}^{min}\left(r,s|a,b\right)\times \left[P\left(r,s|a,b\right)-\rho_{1}^{min}\left(r,s|a,b\right)\right]=0,$$

we have

(68) $$\begin{array}{c} F_{1}^{min}-F^{min}\leq F_{1}^{min}=\\ \sum_{r,s,a,b}W\left(a,b\right)\frac{{\left[P\left(r,s|a,b\right)-\rho_{1}^{min}\left(r,s|a,b\right)\right]}^{2}}{2}\;\\ =\sum_{r,s,a,b}W\left(a,b\right)\frac{P^{2}\left(r,s|a,b\right)-{\left(\rho_{1}^{\min}\right)}^{2}\left(r,s|a,b\right)}{2}\;\\ \leq \sum_{r,s,a,b}W\left(a,b\right)\frac{P^{2}\left(r,s|a,b\right)}{2}\leq \frac{1}{2}. \end{array}$$

Thus, the error decreases at least as fast as 1/n. Although the convergence of the upper bound is sublinear, we derived this inequality by using Equation (63), which provides a quite loose bound on the optimal value $\chi^{min}$. Nonetheless, the constraint set by Equation (66) on the accuracy is strong enough to imply the polynomial convergence of the algorithm, provided that the oracle can be simulated in polynomial time. Indeed, the inequality implies that the number of steps required to reach a given accuracy does not grow faster than ${\left(RS\right)}^{2}$. Since the computational cost of completing each step is polynomial, the overall algorithm has polynomial cost. More precisely, each step is completed by solving a quadratic minimization problem. If we do not rely on the specific structure of the quadratic problem, its computational cost does not grow faster than $\max\{n_{1}^{3},n_{1}^{2}n_{2},D\}$ [19], where $n_{1}$, $n_{2}$, and *D* are the number of variables, the number of constraints, and the cost of evaluating the first and second derivatives of the objective and constraint functions. The numbers $n_{1}$ and $n_{2}$ are equal, and *D* is equal to $n_{1}^{2}\left(A+B\right)$. As the number of vertices in the set $\Omega_{n}$ is not greater than the number of iterations (say, $\bar{n}$), we have that $n_{1}\leq \bar{n}$. Furthermore, the number of vertices cannot be greater than $d_{NS}$. Thus, the number of variables is, in the worst case,

(69) $$n_{1}=\min\left\{\bar{n},ABRS\right\}.$$

As implied by Equation (66), about ${\left(RS\right)}^{2}/\epsilon$ iterations are sufficient for reaching an error not greater than ϵ. Let us set $\bar{n}={\left(RS\right)}^{2}/\epsilon$. Denoting the computational cost of Algorithm 1 with accuracy ϵ by $C_{\epsilon }$, we have that

(70) $$C_{\epsilon }\leq K\bar{n}\max\left\{n_{1}^{3},n_{1}^{2}\left(A+B\right)\right\}=K\frac{{\left(RS\right)}^{2}}{\epsilon }n_{1}^{2}\max\left\{n_{1},A+B\right\},$$

where *K* is some constant. Let us consider the two limiting cases with $\epsilon \left(A+B\right)\geq {\left(RS\right)}^{2}$ (high number of measurements) and $\epsilon ABRS\leq {\left(RS\right)}^{2}$ (high accuracy).

In the first case, we have that $A+B\geq \bar{n}$, which also implies that $n_{1}=\bar{n}$ (there are at least 2 measurements per party). We have

(71) $$\epsilon \left(A+B\right)\geq {\left(RS\right)}^{2}⟹C_{\epsilon }\leq K\frac{{\left(RS\right)}^{6}}{\epsilon^{3}}\left(A+B\right)\equiv B_{0}$$

Thus, given a fixed error, the computational cost is asymptotically linear in the number of measurements. For $\epsilon =10^{-2}$ and R=S=2, this bound holds for a number of measurements per party greater than 800. If A=B=800, the computation ends in few hours in the worst case by using available personal computers, provided that the bound $B_{0}$ is saturated in the most pessimistic scenario.

In the second case, we have that $ABRS\leq \bar{n}$ and $n_{1}=ABRS$. Thus,

(72) $$\epsilon AB\leq RS⟹C_{\epsilon }\leq K\frac{{\left(RS\right)}^{2}}{\epsilon }{\left(ABRS\right)}^{3}\equiv B_{1}.$$

Thus, for a fixed error ϵ and AB smaller than RS/ϵ, the bound on the computational cost scales as the third power of the product AB; that is, the sixth power of the number of measurments, provided that A=B. This scaling is in good agreement with the numerical tests, as discussed later. However the tests indicate that the scaling 1/ϵ and, thus, the sublinear convergence is too pessimistic. For example, for $\epsilon =10^{-3}$, A=B≤40, and R=S=2, the bound gives a running time of the order of months, whereas the running time in the tests turns out to be less than one hour.

### 6.3. Simulation of the Oracle

We have shown that the cost of computing the distance from the local polytope grows polynomially, provided that we have access to the oracle. But what is the computational complexity of the oracle? In the case of measurements with two outcomes, we have seen that the solution of the oracle is equivalent to finding the minimal energy of a particular class of Ising spin glasses. These problems are known to be NP-hard. However, the oracle has a particular structure that can make many physically relevant instances numerically tractable. For example, the couplings of the Ising spin model are constrained by the nonsignaling conditions on P(r,s|a,b) and the optimality conditions (22)–(24). Furthermore, the Hamiltonian (14) is characterized by two classes of spins, described by the variables $r_{k}$ and $s_{k}$, respectively, and each element in one class is coupled only to elements in the other class. This particular structure suggests the following block-maximization algorithm for solving the oracle.

**Algorithm** **2.** *Input: g(r,s;a,b)*

*1.*  *Generate a random sequence r.*
*2.*  *Maximize $\sum_{a,b}g\left(r_{a},s_{b};a,b\right)W\left(a,b\right)$ with respect to the sequence s (see later discussion).*
*3.*  *Maximize $\sum_{a,b}g\left(r_{a},s_{b};a,b\right)W\left(a,b\right)$ with respect to the sequence r.*
*4.*  *Repeat from Step 2 until the block-maximizations stop making progress.*

Numerical tests show that this algorithm, when used for computing the distance from the local polytope, stops after a few iterations. Furthermore, only a few trials of the initial random sequence r are required for convergence of Algorithm 1. We also note that the probability of a successful simulation of the oracle increases when χ is close to the optimal solution $\chi^{min}$, suggesting that the optimality conditions (22)–(24) play some role in the computational complexity of the oracle. Pragmatically, we have chosen the number of trials equal to $d_{NS}$, such that the computational cost of simulating the oracle contributes to the overall running time with a constant multiplicative factor and, thus, the sixth-power law of the oracle-assisted algorithm is not affected.

Before discussing the numerical results, let us explain how the maximization on blocks is performed. Let us consider the maximization with respect to r, as the optimization with respect to s has an identical procedure. We have

(73) $$\begin{array}{c} \max_{r}\sum_{a,b}W\left(a,b\right)g\left(r_{a},s_{b};a,b\right)=\;\\ \sum_{a}\max_{r}\sum_{b}g\left(r,s_{b};a,b\right)W\left(a,b\right)\equiv \;\\ \sum_{a}\max_{r}\tilde{g}\left(r,s;a\right). \end{array}$$

Thus, the maximum is found by maximizing the function $\tilde{g}\left(r,s;a\right)$, with respect to the discrete variable *r* for every *a*. Taking into account the sum over *b* required for generating $\tilde{g}$, the computational cost of the block-maximization is proportional to RAB. Thus, it does not grow more than linearly with respect to the size of the problem input; that is, RSAB.

## 7. Numerical Tests

In the previous sections, we introduced an algorithm that computes the distance from the local polytope in polynomial time, provided that we have access to oracle Max. Surprisingly, in every simulation performed on entangled qubits, the algorithm implementing the oracle successfully finds the solution in polynomial time. More precisely, the algorithm finds a sequence (r,s) sufficiently close to the maximum to guarantee convergence of Algorithm 1 to the solution of Problem 1. Interestingly, the probability of a successful simulation of the oracle increases as χ approaches the solution. This suggests that the optimality conditions (22)–(24) play a fundamental role in the computational complexity of the oracle. To check that the algorithm successfully finds the optimizer $\chi^{min}$ up to the desired accuracy, we have solved the oracle with a brute-force search at the end of the computation, whenever this was possible in a reasonable time. All of the checks show that the solution is found within the desired accuracy.

In the tests, we considered the case of maximally entangled states, Werner states, and pure non-maximally entangled states. The numerical data are compatible with a running time scaling as the sixth power of the number of measurements. This is in accordance with the theoretical analysis, given in Section 6.2. Furthermore, the simulations show that the sublinear convergence of the upper bound $B_{1}$ on the error is very loose, and the convergence turns out to be much faster. Let us discuss the case of entangled qubits in a pure quantum state.

### 7.1. Maximally Entangled State

**Figure 1:**
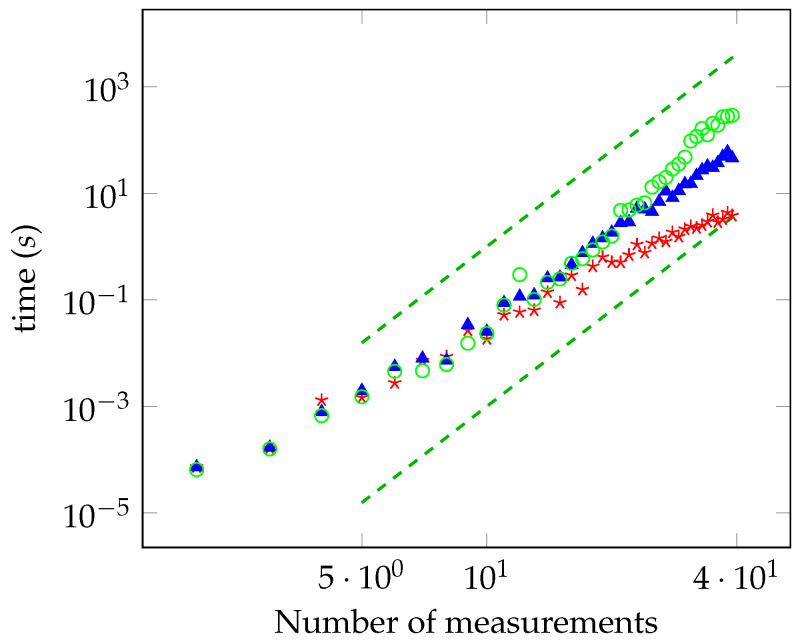
Time required for computing the distance from the local polytope for a maximally entangled state as a function of the number of measurements (log-log scale) with accuracy equal to $10^{-3}$, $10^{-4}$, and $10^{-5}$ (red, blue, and green points, respectively).

In Figure 1, we report the time required for computing the distance from the local polytope as a function of the number of measurements, *M*, in log-log scale. The distance has been evaluated with accuracy equal to $10^{-3}$, $10^{-4}$, and $10^{-5}$ (red, blue, and green points, respectively). We have considered the case of planar measurements on the Bloch sphere. For the sake of comparison, we have also plotted the functions $10^{-6}M^{6}$ and $10^{-9}M^{6}$ (dashed lines). The data are compatible with the theoretical power law derived previously. They also show that the sublinear convergence of $B_{1}$, derived in Section 6.2, is too pessimistic and the algorithm actually shows better performances. In particular, the bound $B_{1}$ says that the running time is not greater than years for A=B=40 and $\epsilon =10^{-5}$, whereas the observed running time is actually less than one hour. Other simulations have been performed with random measurements. We generated a set of measurements corresponding to random vectors on the Bloch sphere, by considering both the planar and non-planar case. Then, we computed the distance from the local polytope for a different number of measurements. We always observed that the running time scales with the same sixth power law. For a number of measurements below 28, we have solved the oracle with a brute-force search at the end of the computation, and we have always found that Algorithm 1 successfully converged to the solution within the desired accuracy.

### 7.2. Non-Maximally Entangled State

In the case of the non-maximally entangled state

(74) $$|\psi \rangle =\frac{|00\rangle +\gamma |11\rangle }{\sqrt{1+\gamma^{2}}},$$

with γ∈[0,1], we have considered planar measurements orthogonal to the Bloch vector ${\vec{v}}_{z}\equiv \left(0,0,1\right)$ (such that the marginal distributions are unbiased), as well as planar measurements lying in the plane containing ${\vec{v}}_{z}$ (biased marginal distributions).

**Figure 2:**
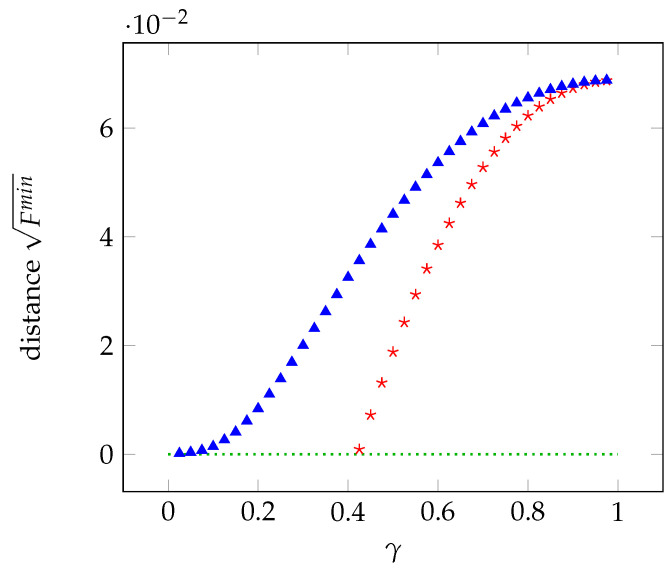
Distance from the local polytope as a function of γ in the unbiased case (red stars) and biased case (blue triangles).

In Figure 2, we report the distance from the local polytope as a function of γ with 10 measurements. The distance changes slightly for higher numbers of measurements. In the unbiased case, the distance goes to zero for γ equal to about 0.4, whereas the correlations become local for γ=0 in the biased case.

**Figure 3:**
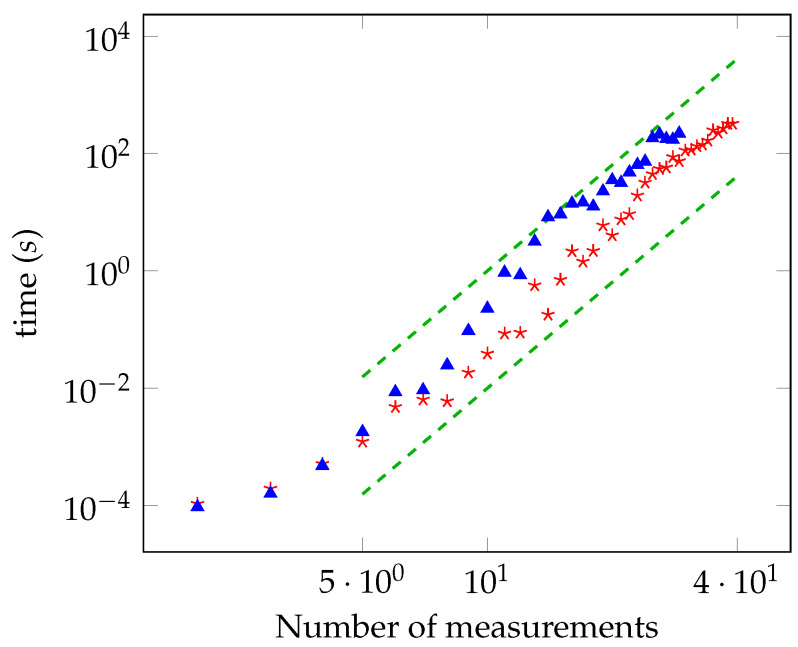
Time required for computing the distance from the local polytope as a function of the number of measurements (log-log scale) in the unbiased case, for γ=0.8 (red stars) and γ=0.6 (blue triangles). The green lines are the functions $10^{-6}M^{6}$ and $10^{-8}M^{6}$. The accuracy is $10^{-5}$.

**Figure 4:**
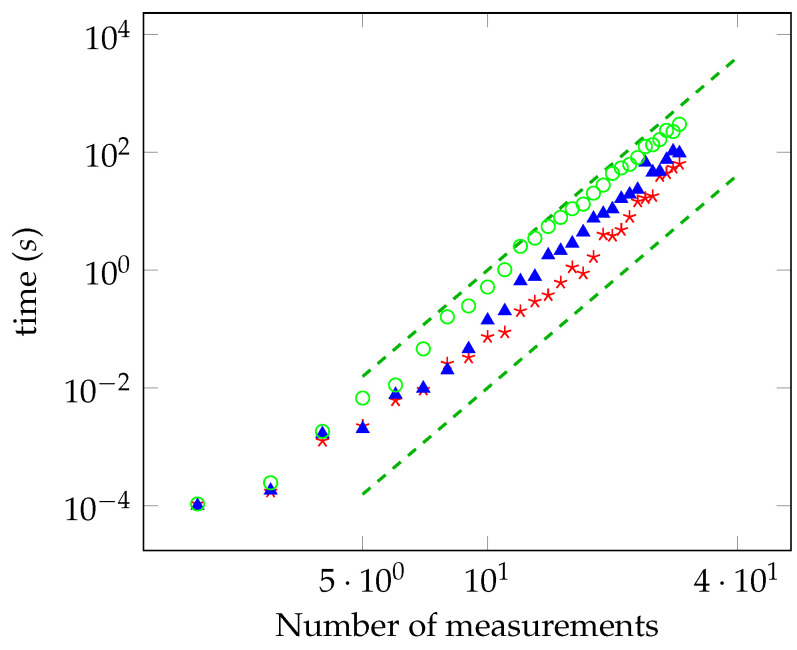
The same as Figure 3 in the biased case, for γ=0.8 (red stars), γ=0.6 (blue triangles), and γ=0.4 (green circles).

In Figure 3 and Figure 4, the running time as a function of the number of measurements is reported for the unbiased and biased cases, respectively. The power law is, again, in accordance with the theoretical analysis. As done for the maximally entangled case, we have checked the convergence to the solution by solving the oracle with a brute force search for a number of measurements up to 28.

## 8. Conclusions

In conclusion, we have presented an algorithm that computes the distance of a given non-signaling box to the local polytope. The running time, with given arbitrary accuracy, scaled polynomially, granted the access to an oracle determining the optimal locality bound of a Bell inequality. We also proposed an algorithm for simulating the oracle. In all of the numerical tests, the overall algorithm successfully computed the distance with the desired accuracy and a scaling of the running time, in agreement with the bound theoretically derived for the oracle-assisted algorithm. Our algorithm opens the way to tackle many unsolved problems in quantum theory, such as the non-locality of Werner states. Since the non-locality problem is NP-hard, our work and its further refinements could provide alternative algorithms to solve some instances of computationally hard problems.
